# Intrinsic Visible Plasmonic Properties of Colloidal PtIn_2_ Intermetallic Nanoparticles

**DOI:** 10.1002/advs.202307055

**Published:** 2024-01-09

**Authors:** Haruka Takekuma, Ryota Sato, Kenji Iida, Tokuhisa Kawawaki, Mitsutaka Haruta, Hiroki Kurata, Katsuyuki Nobusada, Toshiharu Teranishi

**Affiliations:** ^1^ Department of Chemistry Graduate School of Science Kyoto University Gokasho Uji Kyoto 611‐0011 Japan; ^2^ Institute for Chemical Research Kyoto University Gokasho Uji Kyoto 611‐0011 Japan; ^3^ Institute for Catalysis Hokkaido University N21 W10 Kita‐ku Sapporo Hokkaido 001‐0021 Japan; ^4^ Department of Applied Chemistry Faculty of Science Tokyo University of Science 1‐3 Kagurazaka Shinjuku‐ku Tokyo 162‐8601 Japan; ^5^ Research Institute for Science and Technology Tokyo University of Science 1‐3 Kagurazaka Shinjuku‐ku Tokyo 162‐8601 Japan; ^6^ Department of Theoretical and Computational Molecular Science Institute for Molecular Science 38 Nishigonaka, Myodaiji‐cho Okazaki Aichi 444‐8585 Japan

**Keywords:** intermetallic phase, nanoparticles, non‐coinage metal, screening effect, surface plasmon resonance

## Abstract

Materials that intrinsically exhibit localized surface plasmon resonance (LSPR) in the visible region have been predominantly researched on nanoparticles (NPs) composed of coinage metals, namely Au, Ag, and Cu. Here, as a coinage metal‐free intermetallic NPs, colloidal PtIn_2_ NPs with a *C*1 (CaF_2_‐type) crystal structure are synthesized by the liquid phase method, which evidently exhibit LSPR at wavelengths similar to face‐centered cubic (*fcc*)‐Au NPs. Computational simulations pointed out differences in the electronic structure and photo‐excited electron dynamics between *C*1‐PtIn_2_ and *fcc*‐Au NPs; reduces interband transition and stronger screening with smaller number of bound *d*‐electrons compare with *fcc*‐Au are unique origins of the visible plasmonic nature of *C*1‐PtIn_2_ NPs. These results strongly indicate that the intermetallic NPs are expected to address the development of alternative plasmonic materials by tuning their crystal structure and composition.

## Graphical abstract

Colloidal PtIn_2_ intermetallic NPs with a *C*1 (CaF_2_‐type) crystal structure clearly exhibit localized surface plasmon resonance (LSPR) in visible region similar to face‐centered cubic (*fcc*) Au NPs. Computational simulations reveal that the visible LSPR is derived from the crystal structure‐dependent electron dynamics and the electronic structure similar to those of coinage metals.

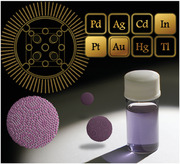

## 1 Introduction

Localized surface plasmon resonance (LSPR) is the collective oscillation of free carriers in conductive nanostructures with a size comparable to or smaller than the wavelength of incident light^[^ ^1^, ^2^, ^3^, ^4^ ^]^ and is an active field of research in areas such as biology,^[^ ^5^, ^6^ ^]^ photochemistry,^[^ ^7^, ^8^ ^]^ nano‐optics,^[^ ^9^, ^10^ ^]^ and art.^[^ ^11^, ^12^ ^]^ This research interest is attributable to electric field enhancement, wavelength‐tunable carrier transfer, and vivid color development, among others. A specific variety of nanomaterials [such as metals (e.g., Pd, Ag, and Au),^[^ ^1^, ^2^, ^3^ ^]^ semiconductors (e.g., indium tin oxide, Al‐doped zinc oxide, and copper‐deficient copper chalcogenides),^[^ ^13^, ^14^ ^]^ and nitrides of group 4 elements (TiN, ZrN, and HfN)^[^ ^15^, ^16^, ^17^ ^]^ have been investigated for plasmonic properties and applications in the ultraviolet (UV) to near‐infrared (NIR) region. It is a well‐known fact that the shape, size, and composition of these nanomaterials can be controlled to further tune the LSPR wavelength.^[^ ^18^, ^19^, ^20^ ^]^ Such research in the visible region is especially important because visible light accounts for half of the available and useful solar energy. For more than 160 years since Faraday solved a mystery of the red color of Au nanoparticles (NPs) colloidally dispersed in a medium,^[^ ^3^ ^]^ researchers have reported that as spherical NPs, only coinage metals (Au, Ag, and Cu) and their alloys clearly exhibit intrinsic LSPR in the visible region. Recent research indicates that Al and Pd anisotropic NPs exhibit visible LSPR,^[^ ^21^, ^22^ ^]^ but spherical NPs of these metals intrinsically exhibit LSPR in the UV region.^[^ ^21^, ^22^, ^23^ ^]^ Most research on visible LSPR continues to focus on coinage‐metal‐based alloys because of their facile synthesis and high chemical stability.^[^ ^24^, ^25^ ^]^ For example, Schaak et al. found that the LSPR wavelength of Au NPs blue‐shift by forming Au‐rich Au–Zn ordered alloys^[^ ^24^ ^]^; Amendola et al. reported a similar blue‐shift of the LSPR wavelength by alloying Au NPs with Fe.^[^ ^25^ ^]^ These studies discussed a modulation of coinage metal NPs, and no detailed research has focused on spherical alloy NPs that do not contain coinage metals yet intrinsically exhibit LSPR in the visible region. Although coinage metals have advantages in LSPR (such as high quality factor for Ag^[^ ^2^ ^]^ and high chemical stability for Au^[^ ^1^, ^2^ ^]^), these limited metals and their combinations make it difficult to overcome their serious drawbacks: facile oxidation of Ag and Cu, and high cost for Au. Determining the principles of visible LSPR in alloy NPs that do not comprise coinage metals would dramatically expand the LSPR library and facilitate discovery of NPs that are far superior to conventional plasmonic NPs (in terms of physical and chemical properties).

Here, we demonstrate that the coinage metal‐free PtIn_2_ colloidal NPs with a *C*1 (CaF_2_‐type) crystal structure, which are difficult to synthesize as a single phase, clearly exhibit LSPR at wavelengths similar to face‐centered cubic (*fcc*)Au NPs. As computational simulations pointed out differences in the electronic structure and photo‐excited electron dynamics between *C*1‐PtIn_2_ and *fcc*‐Au NP, reduced interband transition and stronger screening with smaller number of bound *d*‐electrons compared with *fcc*‐Au are unique origins of the visible plasmonic nature of *C*1‐PtIn_2_ NPs.

## 2 Results and Discussion

### 2.1 Design and Synthesis of PtIn_2_ Intermetallic NP

**Figure 1:**
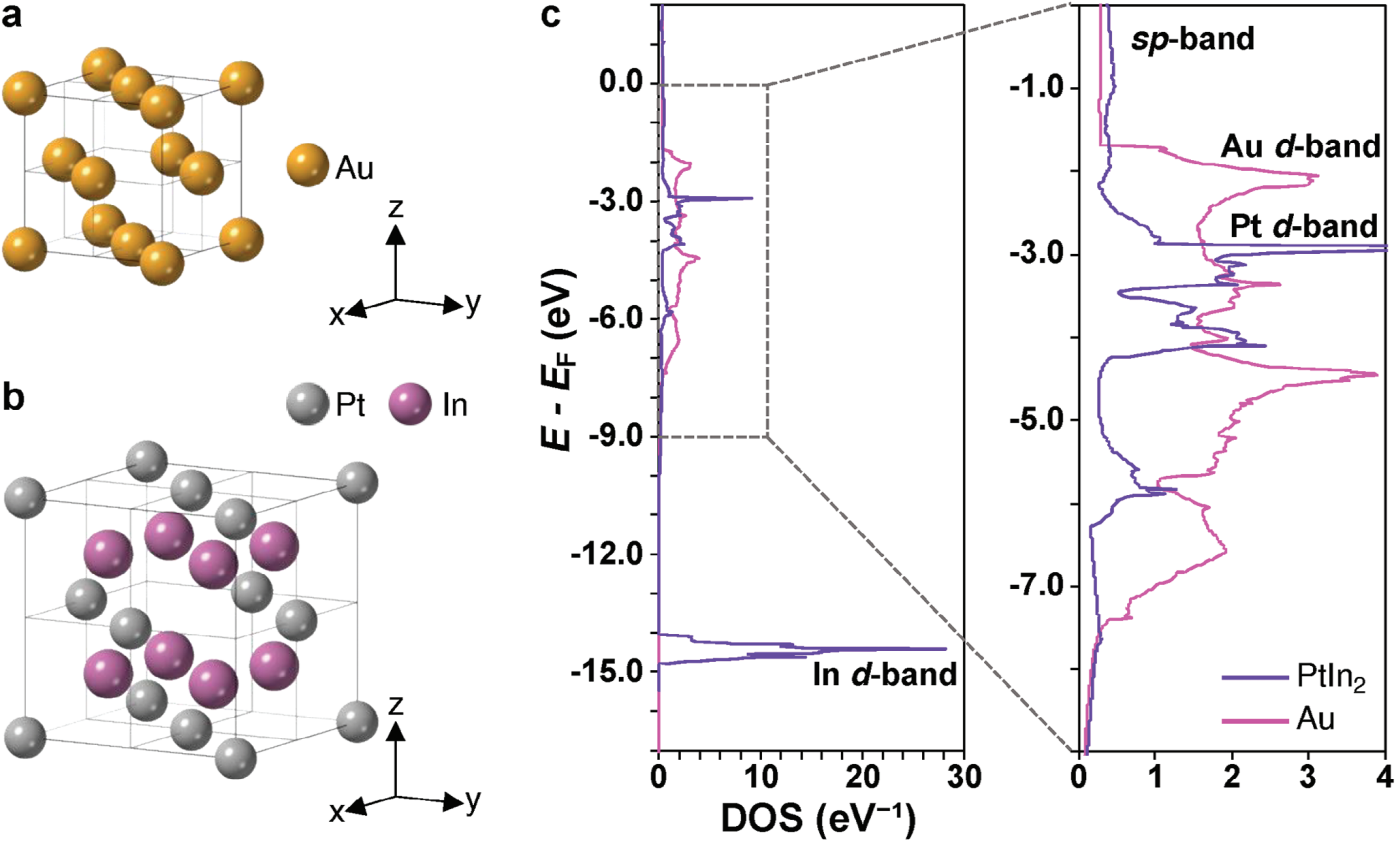
Crystal structures of a) *fcc*‐Au and b) *C*1‐PtIn_2_. c) Calculated density of states (DOS) of bulk *fcc*‐Au (pink) and *C*1‐PtIn_2_ (violet) plotted against energy versus Fermi energy (*E*_F_), adapted from Ref. [27]

What causes LSPR in the visible region? Recent theoretical studies on photo‐excited electron dynamics in Au NPs indicate the cause as not only the interband transitions but also screening caused by the vibration of bound electrons by visible light.^[^ ^4^ ^]^ Thus, we focused on alloy NPs that fulfil two requirements: (i) crystal structure substantially differs from the *fcc* structure in terms of the coordination environment of the atoms, and (ii) electronic structure similar to that of coinage metals. We chose PtIn_2_ intermetallic NPs with a *C*1 (CaF_2_‐type) structure as uncommon types of visible plasmonic NPs. **Figure** **1** shows the crystal and electronic structures of *fcc*‐Au and *C*1‐PtIn_2_. *C*1‐PtIn_2_ has a cubic crystal system like *fcc*‐Au, but non‐close packed structure (Figure 1a,b). Their electronic structures show similar *sp*‐bands crossing the Fermi level and different *d*‐band edges around the visible region (≈−1.7 eV for Au and approximately −2.5 eV for PtIn_2_; PtIn_2_ has an In *d*‐band at ca. −14.5 eV).

We used liquid‐phase chemical synthesis to obtain quasi‐spherical PtIn_2_ NPs several tens of nanometers in diameter for clear observation of LSPR. To avoid oxidation of typical elements such as In, He et al. used typical element–amide complexes as precursors to obtain NPs in the absence of oxygen‐containing molecules.^[^ ^26^ ^]^ We synthesized *C*1‐PtIn_2_ NPs by combining this method with seeded growth. Specifically, we reacted InCl_3_ with oleylamine under strong base conditions, and In–oleylamide complexes (generated in situ) with Pt seed NPs in an inert gas atmosphere, to synthesize *C*1‐PtIn_2_ NPs of various sizes (see Supporting Information for detailed synthetic procedures and size separation of *C*1‐PtIn_2_ NPs).

**Figure 2:**
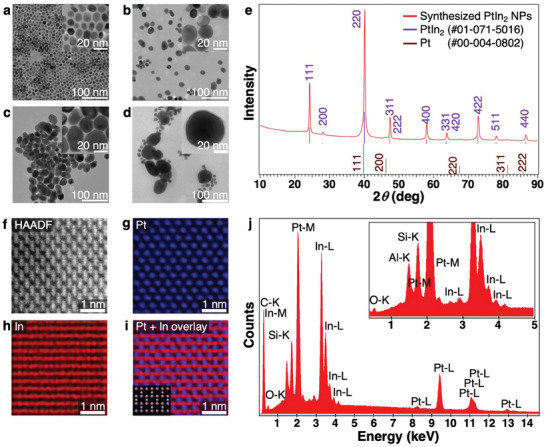
TEM images of a) 10 ± 1 nm Pt seed NPs, b) 17 ± 5, c) 24 ± 8, and d) 44 ± 12 nm PtIn_2_ NPs. The size‐distribution histograms are shown in Figure S2 (Supporting Information). e) XRD pattern of 44‐nm PtIn_2_ NPs and the standard XRD patterns of PtIn_2_ (violet, PDF #01‐071‐5016) and Pt (brown, PDF #00‐004‐0802). Figure S1 shows more‐detailed XRD analysis data from synchrotron XRD measurements. f) HAADF‐STEM imaging of 44‐nm‐PtIn_2_ NPs, g) Pt element mapping (blue), h) In element mapping (red), and i) element mapping overlay for Pt and In. The inset of the overlay image shows the lattice of *C*1‐PtIn_2_ observed from <110> (Pt atoms: gray, In atoms: pink). j) SEM‐EDX spectrum of *C*1‐PtIn_2_ NPs showing peaks corresponding to Pt, In, Si, Al, and O, where O is coming from the sample and quartz substrate and Si and Al are from the quartz substrate and the sample stage of SEM. Pt:In:O = 30.7:46.8:22.5 (at%).

We separated the PtIn_2_ NPs synthesized from 10 ± 2 nm Pt seed NPs (**Figure** **2**a) at 180 °C by centrifugation into two fractions (Figure 2b,c), the sizes of which were 17 ± 5 and 24 ± 8 nm, respectively. PtIn_2_ NPs that synthesized at 240 °C exhibited a drastic increase in size (44 ± 12 nm) with a broad size distribution (Figure 2d). Because a single PtIn_2_ NP increases in size by 1.6× compared with a Pt seed NP by an estimation of the unit cell change, it is clear that the NPs grew by interparticle fusion during the high‐temperature reaction. The roundness of the particles in Figure 2d was 0.84 ± 0.07, indicating that we could obtain quasi‐spherical large PtIn_2_ NPs to clearly observe the LSPR. Figure 2e shows the X‐ray diffraction (XRD) pattern of as‐synthesized 44‐nm PtIn_2_ NPs. Rietveld refinement of this diffraction pattern indicates that the NPs comprised a *C*1‐PtIn_2_ phase as a main phase (92.6 wt%) with smaller *fcc*‐Pt‐based crystallites as a minor phase (7.4 wt%) (Figure S1, Supporting Information). These transmission electron microscopy (TEM) and XRD results indicate that the smaller and larger NPs in Figure 2d correspond to the Pt phase slightly alloyed with In and the *C*1‐PtIn_2_ phase, respectively. The atomic ratio (Pt : In = 1:1.49) calculated by the Rietveld refinement is also in reasonable agreement with the atomic ratio (Pt : In = 1:1.52) measured by scanning electron microscopy‐energy‐dispersive X‐ray spectroscopy (SEM‐EDX) analysis (Figure 2j). Atomic resolution analytical TEM observation revealed the ordered crystal structure of 44‐nm PtIn_2_ NPs (Figure 2f–i). A high‐angle annular dark field scanning TEM (HAADF‐STEM) image shows the lattice observed from <110> and TEM‐EDX measurements of Pt and In confirmed the *C*1 structure. Both macroscopic measurements by XRD and microscopic observations by atomic resolution analytical TEM proved the *C*1 structure of synthesized NPs. Our synthesis gave the *C*1‐PtIn_2_ phase, a high‐temperature‐stable phase in bulk (Figure S3, Supporting Information), even at a considerably lower temperature than the phase transition temperature (674 °C).^[^ ^27^ ^]^ Such a phenomenon is common in d‐phase synthesis of inorganic NPs and is caused by thermodynamic effects in phase formation and kinetic effects in atomic diffusion as the specific surface area of the NPs increases.^[^ ^28^, ^29^ ^]^

### 2.2 Visible Plasmonic Features of *C*1‐PtIn_2_ NPs

**Figure 3:**
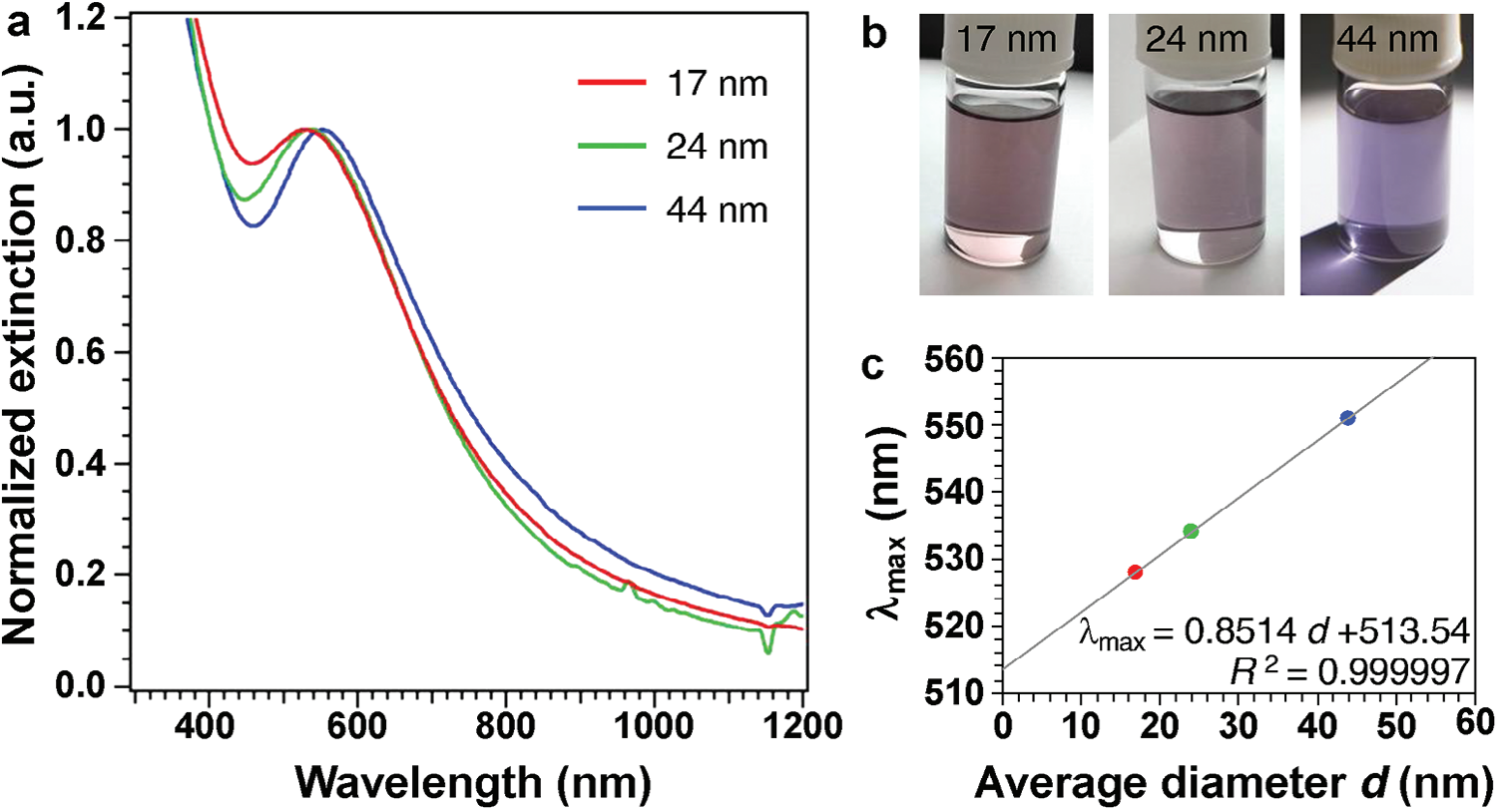
a) UV–vis spectra and b) photos of PtIn_2_ NPs with sizes of 17, 24, and 44 nm; corresponding to Figure 2b,c,d, respectively. Their LSPR extinction maximum wavelengths (*λ*_max_) were 528, 534, and 551 nm, respectively. Chloroform dispersions of purified 17‐ and 44‐nm *C*1‐PtIn_2_ NPs were reddish‐purple and vivid violet, respectively. c) Average diameter versus *λ*_max_. The *λ*_max_ was proportional to the average diameter of the NPs (*d*), which is characteristic of LSPR.

Surprisingly, a chloroform dispersion of purified 44‐nm *C*1‐PtIn_2_ NPs was bright violet and its UV–vis spectrum indicated an extinction maximum wavelength (*λ*_max_) at 551 nm (2.25 eV), suggesting that the *C*1‐PtIn_2_ structure is responsible for this violet color development (**Figure** **3**a,b). Note that Pt‐seed NPs showed no LSPR peak in the visible region (Figure S4, Supporting Information). The *λ*_max_ values of 17‐ and 24‐nm PtIn_2_ NPs at 528 nm (2.35 eV) and 534 nm (2.32 eV), respectively, indicate that the peak position red‐shifted with increasing particle size (Figure 3c), which is characteristic of LSPR.^[^ ^19^ ^]^ The *λ*_max_/nm values of spherical 12–50 nm Ag NPs and 15–48 nm Au NPs were 0.68^[^ ^30^ ^]^ and 0.41,^[^ ^31^ ^]^ respectively. Compared with these coinage metal NPs, PtIn_2_ NPs exhibited a larger *λ*_max_/nm value of 0.85. In addition, the UV–vis spectra of 17‐nm PtIn_2_ NPs in cyclohexane (refractive index: *n* = 1.4268), chloroform (*n* = 1.4467), and toluene (*n* = 1.4978) indicate that the *λ*_max_ was proportional to the refractive index of the solvent (Figure S5, Supporting Information), which also supports the LSPR feature of *C*1‐PtIn_2_ NPs. We calculated the sensitivity of LSPR *λ*_max_ to refractive index unit (RIU) to be *λ*_max_/RIU = 42.4, which is closer to that of 25‐nm Au NPs (*λ*_max_/RIU = 78)^[^ ^32^ ^]^ rather than that of 25‐nm Ag NPs (*λ*_max_/RIU = 163).^[^ ^32^ ^]^ Because these peak shifts are well known in plasmonic NPs, we assign the extinction peaks of *C*1‐PtIn_2_ NPs to LSPR. Atomic resolution analytical TEM observations showed the double‐shells around the *C*1‐PtIn_2_ cores (Figures S6 and S7, Supporting Information). During the purification and preservation, zero‐valent In atoms in the outermost surface of PtIn_2_ NPs might be gradually oxidized and excluded to form indium oxide layers as outer shells and Pt‐rich layers as inner shells (ca. 1 nm). This double‐shell structure may prevent further oxidation of PtIn_2_ NPs, as passive state film, and maintain LSPR properties for a long time. Furthermore, this double‐shell structure may contribute to the larger *λ*_max_/nm value and the lower refractive index sensitivity than Au NPs; expanding the library of plasmonic intermetallic NPs will clarify the mechanism for this phenomenon.

**Figure 4:**
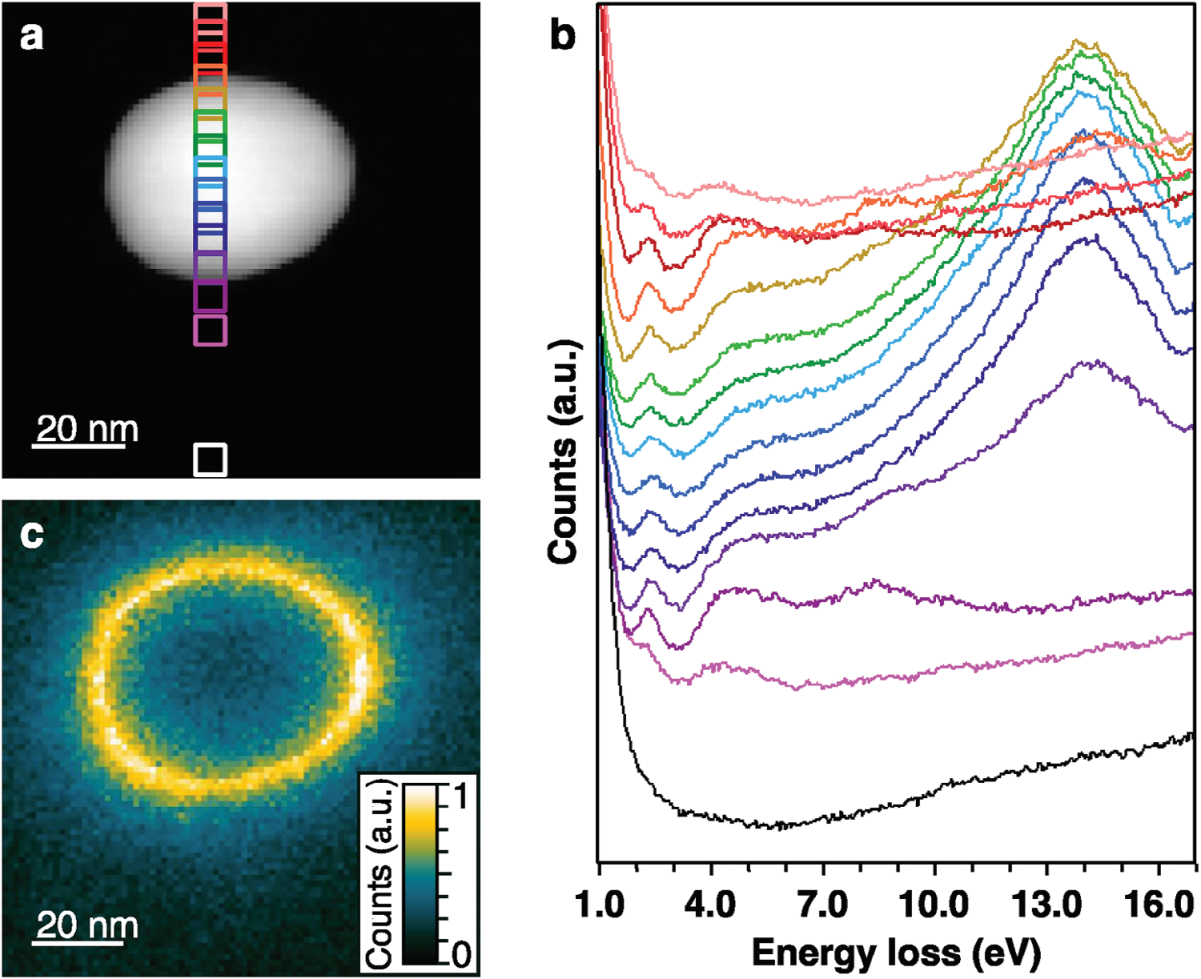
a) HAADF‐STEM image of a 40‐nm PtIn_2_ NP. b) Series of 15 STEM‐EEL spectra corresponding to the rectangular regions in (a). The colors of the squares in (a) correspond to the colors of the spectrum in (b), but only the white squares are shown with black lines. We detected two main resonance positions along the line scan at 2.3 and 14.2 eV. c) EELS map of the PtIn_2_ NP at 2.1–2.5 eV (centered at 2.3 eV). The energy loss at 2.3 eV occurred at the NP surface, which clearly indicates that PtIn_2_ NPs exhibit LSPR.

To further confirm the visible LSPR feature of *C*1‐PtIn_2_ NPs, we conducted scanning TEM‐electron energy loss spectroscopy (STEM‐EELS) analysis of a single *C*1‐PtIn_2_ NP as one of the most powerful techniques to demonstrate LSPR.^[^ ^33^ ^]^ To prevent contamination from organic ligands, the SiN membrane was heated at 400 °C in the STEM experiments. **Figure** **4**a shows a HAADF‐STEM image of an ≈40‐nm single PtIn_2_ NP. The electron energy loss spectra collected from rectangular regions aligned across the diameter (Figure 4b) indicate that the peak intensities at 2.3 eV (539 nm) that are characteristic of LSPR (2.25 eV, 551 nm, Figure 3a) at the surface of the NP were higher than those at the center of the NP. EELS mapping of a single PtIn_2_ NP at 2.1–2.5 eV indicate the highest excitation probability at the surface (Figure 4c). We observed the shoulder peaks at 4.9 and 8.4 eV with relatively higher intensities at the surface, and the high excitation probability at the NP surface in the EELS mappings at 4.7–5.1 and 8.2–8.6 eV (Figure S8a,b, Supporting Information). Because in the case of an interband transition there should be an energy loss over the entire NP, the EELS peaks at 4.9 and 8.4 eV could be assigned to high‐order LSPR modes, such as a quadrupole mode, or surface exciton polariton.^[^ ^34^ ^]^ In addition, the peaks at ≈14.2 eV in Figure S8c (Supporting Information) can be attributed to the interband transition from the localized In *d*‐band to the Fermi level and/or the bulk plasmon (volume plasmon), which are collective free electron oscillations in compressional waves inside the NP caused by electron beams, corresponding to the highest excitation probability at the center of the NP.

We conducted TEM observations of the same NP at 400 °C after the EELS measurements to confirm preservation of the *C*1‐PtIn_2_ phase even under high‐temperature conditions. We clearly observed the lattice fringe throughout the NP (Figure S9a,b, Supporting Information), and a fast‐Fourier transform (FFT) image of this lattice fringe (Figure S9c, Supporting Information) indicates spots corresponding to the 200, 220, and 400 reflections of the *C*1 structure. The noise‐filtered inverse FFT indicates an ordered pattern (Figure S9d, Supporting Information), which is consistent with the {100} plane of *C*1‐PtIn_2_. In addition, we also confirmed the *C*1‐PtIn_2_ phase stability at 400 °C by in situ high‐temperature XRD (Figure S10, Supporting Information).

### 2.3 Plasmonic Functionality of *C*1‐PtIn_2_ NPs

To demonstrate plasmonic properties of *C*1‐PtIn_2_ NPs, we measured surface‐enhanced Raman scattering (SERS) of an organic dye, rhodamine 6G (R6G), as a probe molecule. For comparison, we synthesized and used 17‐nm Au NPs for SERS measurements of R6G (Figure S11, Supporting Information). We detected no clear peaks assigned to R6G from R6G without NPs, whereas we observed strong and sharp peaks in the presence of *C*1‐PtIn_2_ and Au NPs (Figure S12, Supporting Information). The SERS enhancement by *C*1‐PtIn_2_ NPs was less than half that by Au NPs, comparable to our prediction of the corresponding quality factors (*Q*‐factors): 2.80 and 7.98, estimated from the extinction spectra of PtIn_2_ and Au NPs, respectively (Figure S11, Supporting Information). Researchers do not fully understand to what extent this enhancement is due to electromagnetic and chemical mechanisms.^[^ ^35^ ^]^ Polydispersity, aggregation, and large size of PtIn_2_ NPs are considered to increase full width half maximum of the UV–vis spectra and reduce the *Q*‐factors. In addition, we propose the *Q*‐factors in the LSPR of ordered alloy NPs should be affected by the degree of order, crystalline size (or grain size), and surface elemental compositions.

The phase diagram (Figure S3, Supporting Information) shows that the PtIn_2_ phase is a line compound with a very narrow composition range, which indicates that PtIn_2_ has a high degree of order. TEM‐EDX measurements confirmed the higher crystalline *C*1 structure (Figure 2g–i). Moreover, since the experimental result (Figure S1, Supporting Information) was similar to the simulation pattern with high Pt occupancy at Pt sites (Figure S14a, Supporting Information), the obtained PtIn_2_ is considered to have a highly‐ordered crystal structure. Therefore, the *Q*‐factor degradation due to the disordered structure is negligible. The HAADF‐STEM observation did not confirm the clear grain boundary of PtIn_2_, but showed the double‐shell structure (Figures S6 and S7, Supporting Information), which prevents further oxidization but increase LSPR damping. This would reduce the *Q*‐factor and the Raman peak intensity.

As the *Q*‐factor of PtIn_2_ NPs was larger than those of spherical and highly dispersed metal oxide‐based semiconductors such as WO_3−_ *_x_* NPs (1.5, *λ* = 613 nm),^[^ ^36^ ^]^ ReO_3_ NPs (2.4, *λ* = 590 nm),^[^ ^37^ ^]^ and ZnO:Sn NPs (1.4, *λ* = 790 nm),^[^ ^38^ ^]^ which indicates that *C*1‐PtIn_2_ is a promising material. The purer, more monodisperse, and highly crystalline *C*1‐PtIn_2_ NPs could provide the larger intrinsic *Q*‐factor. We would like to emphasize that visible plasmonic alloy NPs without any coinage metals can be created on the basis of their electronic and crystal structures.

### 2.4 Difference in Photo‐Excited Electron Dynamics Between *C*1‐PtIn_2_ and *fcc*‐Au NPs

**Figure 5:**
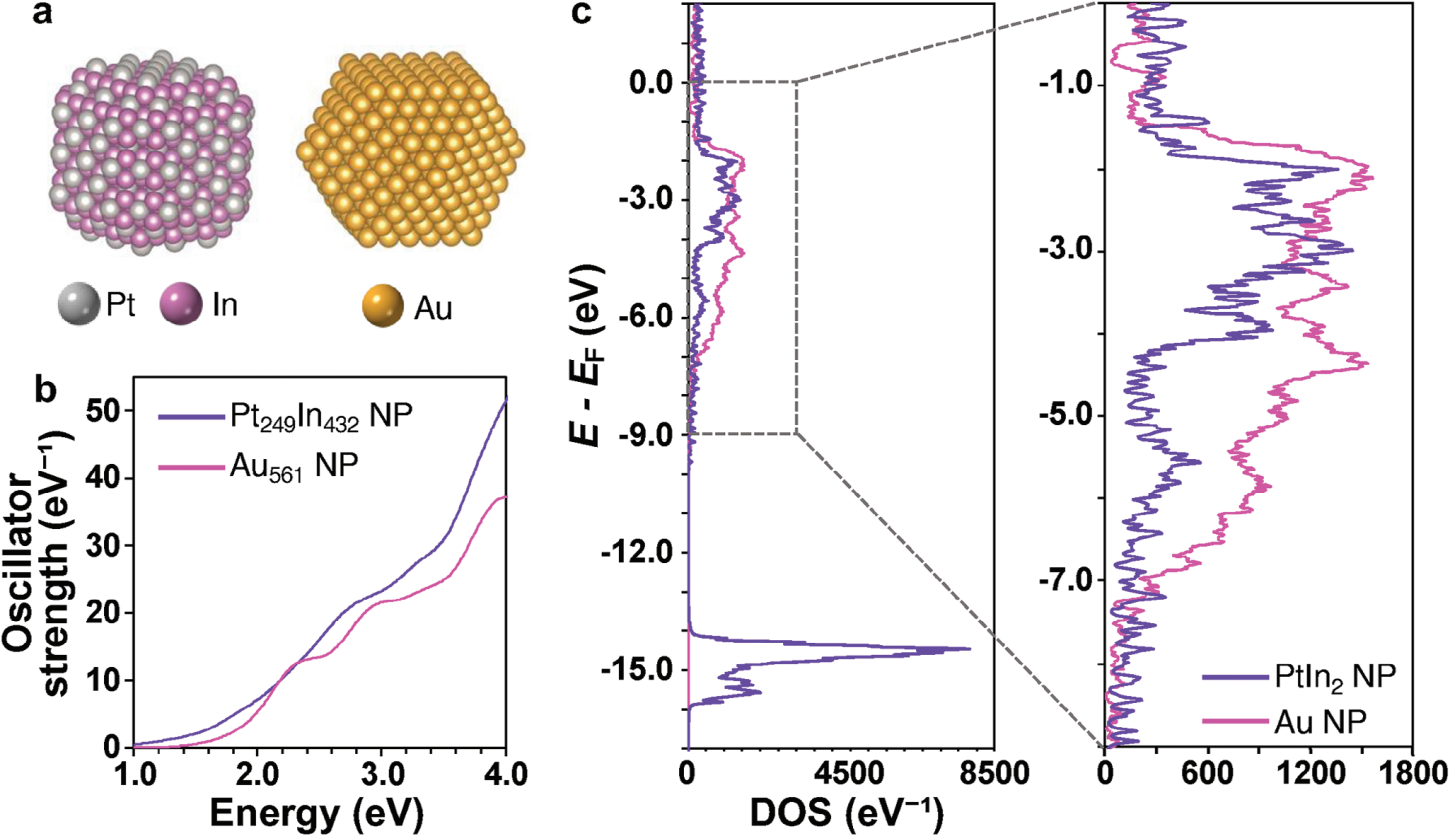
a) Models of *C*1‐Pt_249_In_432_ and *fcc*‐Au_561_ NPs. Because we cut both faceted NPs with a given number of constituent atoms from bulk crystals, the shapes, and sizes varied depending on the crystal structure, and the PtIn_2_ was more In‐rich than in an ideal ratio. b) Calculated oscillator strength curves of Au_561_ (pink) and *C*1‐Pt_249_In_432_ (violet) NPs. c) Calculated density of states (DOS) plotted against energy with the Fermi energy (*E*_F_) of Au_561_ (pink) and *C*1‐Pt_249_In_432_ (violet) NPs. In contrast to the bulk DOS (Figure 1), they exhibited similar *d*‐band edges.

Finally, we theoretically investigated the detailed mechanism by which *C*1‐PtIn_2_ NPs exhibit LSPR in the visible region with a first‐principles computational program: SALMON. We performed computational simulations of *C*1‐PtIn_2_ and *fcc*‐Au with nanoparticulate models consisting of ca. 600 atoms, Pt_249_In_432_ and Au_561_ (**Figure** **5**a). In other words, the numbers of Pt and In atoms are roughly one‐third and two‐thirds of the number of Au atoms, respectively, and this approximation can be applied to *sp* and *d* electrons as well. The model size of ca. 2.5 nm we employed is large enough to discuss the LSPR of larger NPs.^[^ ^4^ ^]^ The oscillator strength curves (corresponding to the absorption spectra) of Pt_249_In_432_ and Au_561_ NPs (Figure 5b) exhibit peaks that we assigned to LSPR in both Pt_249_In_432_ and Au_561_ at ca. 2.8 and 2.3 eV, respectively. In both Pt_249_In_432_ and Au_561_ NPs, the collective resonance can be excited because free electrons in the broad *sp*‐band near the Fermi level are present, based on the calculated DOS (Figure 5c). The *d*‐band edge of Pt_249_In_432_ was calculated to be ≈−1.5 eV higher than that of the bulk PtIn_2_ (Figure 1c), which would be derived from the size and shape of the Pt_249_In_432_ model, as well as to the elemental species on the topmost surface. The *d*‐electrons of Pt and Au are mainly distributed in a slightly low energy range relative to the Fermi level, specifically from −7 to −1.5 eV. These electronic structure profiles are similar in Pt_249_In_432_ and Au_561_, and enable an interband transition in the visible‐light range. However, we found an apparent difference in that the In *d*‐electrons are located in a much lower‐energy region of ca. −15 eV in Pt_249_In_432_ NPs and do not contribute to an interband transition by visible‐light irradiation. Damping of the collective electron oscillation occurs due to the interband transition and causes low LSPR intensity. Therefore, it is reasonable for *C*1‐PtIn_2_ with less damping property, to impart a sufficiently high LSPR intensity.

**Figure 6:**
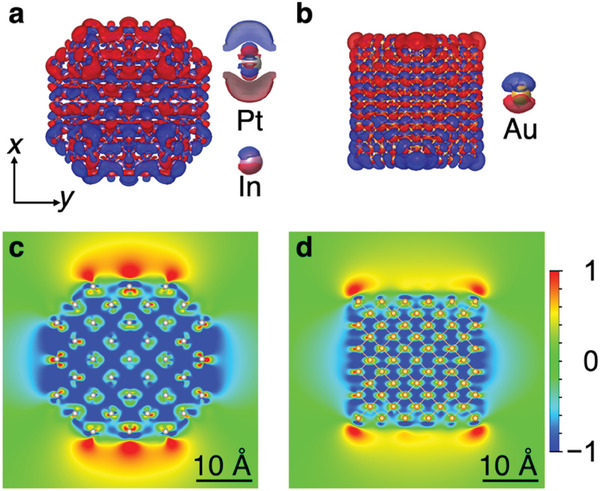
Photo‐induced electron densities of a) *C*1‐Pt_249_In_432_ and b) *fcc*‐Au_561_ NPs observed from the direction perpendicular to the *x*–*y* plane. We applied 2.8‐ and 2.5‐eV *x*‐polarized laser pulses to Pt_249_In_432_ and Au_561_ NPs, respectively. Red and blue represent an increase and a decrease, respectively, in the electron density from the ground state. The equally enlarged atoms on the right side of the overall NPs in (a,b) are cross‐sectional view in the *x*–*y* plane and exhibit the screening effect. Photo‐induced electric field maps of c) Pt_249_In_432_ and d) Au_561_ NPs observed from the direction perpendicular to the *x*–*y* plane. We applied 2.8‐eV *x*‐polarized light to Pt_249_In_432_ and Au_561_ NPs. The distributions of the *x* component with values larger than the threshold value of 1 are represented by the same color (i.e., red) for the maximum values. Similarly, the distributions smaller than −1 are represented in blue. The overall NPs in (a–d) are all drawn at the same scale.

The role of bound *d*‐electrons is not limited to the reduction of the LSPR intensity. The resonance energy of nanoscale coinage metals substantially decreases by screening of the *d*‐electrons.^[^ ^4^, ^39^ ^]^ This is because the polarization of the *d*‐electrons reduces the electric field created by the collectively oscillating free electrons. In *C*1‐PtIn_2_ NPs, the In *d*‐electrons are tightly bound to the nucleus; thus, the number of Pt *d*‐electrons that are responsible for the screening is only ca. one‐third that of coinage metal NPs. From this fact, it is expected that the screening of *C*1‐PtIn_2_ is weaker than that of coinage metal NPs and that the resonance energy does not decrease as much as those of coinage metal NPs do. However, in practice, LSPR of *C*1‐PtIn_2_ NPs occurs in the visible range. We clarified this mystery regarding the LSPR energy of *C*1‐PtIn_2_ NPs by analyzing the photo‐excited electron dynamics at the atomic scale. **Figure** **6**a shows the imaginary part of the photo‐induced electron density change by a 2.8‐eV laser pulse. Positive and negative electron densities are distributed in the top and bottom sides of the NP surface along the laser polarization direction. We attribute the particularly strong polarization around the particle surface to the collective electron motion of the LSPR excitation. The electrons bound to the nuclei contribute to the screening. The electron densities around the atoms (top in blue, bottom in red) are in the opposite phases to the electron densities on the NPs surface (top in red, bottom in blue). Figure 6c shows the photo‐induced electric field, in which we observed the small field enhancement due to a small NP model. Wide green areas around atoms inside the particle are generated by the oscillation of electrons in anti‐phase to that around the surface and are attributable to the oscillation of bound electrons with a large displacement centering around the Pt atoms; that is, the screening occurs mainly by the strongly polarized *d*‐electrons of Pt (Figure 6a,c). This behavior considerably differs from that of coinage metal NPs, in which weak *d*‐electron polarizations are in the close proximity to each nucleus (Figure 6b,d). The large electron displacement (namely, the strong polarization) inside the particle substantially reduces the resonance energy. On the basis of the aforementioned discussions related to the interband transition and screening, our computational simulations reveal that the main origins of the visible plasmonic features in *C*1‐PtIn_2_ NPs are as follows: (1) reduction in the interband transitions and (2) strong screening with a small number of bound *d*‐electrons.

Consequently, we can extract the design principles of intermetallic NPs that exhibit LSPR in the visible region in terms of the crystal and electronic structures as follows. (i) The electronic structure of intermetallic NPs should be similar to that of group 11 element NPs, that is, the *sp*‐band crossing the Fermi level and *d*‐band edge of noble metal like Pt around the visible region. The deeper *d*‐band edge of another base metal like indium less affects the LSPR property. (ii) The intermetallic NPs should have non‐close packed structure to cause strong screening effect by a small number of *d*‐electrons of noble metal. The intermetallic NPs, which satisfy the above two requirements, are expected to show LSPR in the visible region and other candidates will be presented in the forthcoming papers.

## 3 Conclusion

We found that these features are quite similar with the intrinsically metallic electronic structure of group 4 metal nitrides with *B*1 (NaCl‐type) crystal structure, that is, the existence of both delocalized metal *d*‐band near the Fermi level and localized N 2*p*‐band at several eV below *E*_F_.^[^ ^40^ ^]^ We also found that the crystal structure has a significant effect on the photo‐excited electron dynamics, especially on the screening to reduce the electric field. Therefore, we strongly expected that a variety of ordered alloy NPs can precisely modulate the LSPR properties in the wide spectral range as alternative candidates for plasmonic materials by coordinating the crystal structure, such as atomic arrangement, composition, ordering degree, etc. This study would contribute to understanding of the LSPR properties of ordered alloy NPs and enrich the library of plasmonic nanomaterials.

## Conflict of Interest

The authors declare no conflict of interest.

## Supporting information

Supporting Information

## Data Availability

The data that support the findings of this study are available in the supplementary material of this article.
