# Supplementary material for: Intrinsic Visible Plasmonic Properties of Colloidal PtIn2 Intermetallic Nanoparticles
Source: Adv Sci (Weinh). 2024 Jan 9;11(10):2307055. doi: 10.1002/advs.202307055 (PMC10933610; doi:10.1002/advs.202307055)
Supplement: Supplementary file 1 — Supporting Information [file ADVS-11-2307055-s001.pdf]

ADVANCED SCIENCE
Open Access

Supporting Information

for *Adv. Sci.*, DOI 10.1002/advs.202307055

Intrinsic Visible Plasmonic Properties of Colloidal $PtIn_2$ Intermetallic Nanoparticles

Haruka Takekuma, Ryota Sato, Kenji Iida, Tokuhisa Kawawaki, Mitsutaka Haruta, Hiroki Kurata, Katsuyuki Nobusada and Toshiharu Teranishi**

Supporting Information

Intrinsic Visible Plasmonic Properties of Colloidal $PtIn_2$ Intermetallic Nanoparticles

Haruka Takekuma, Ryota Sato,* Kenji Iida, Tokuhisa Kawawaki, Mitsutaka Haruta, Hiroki Kurata, Katsuyuki Nobusada, Toshiharu Teranishi*

General experimental information

All the solvents and reagents were commercially available. Platinum acetylacetonate [$Pt(acac)_2$, 97%]; lithium diisopropylamide (LDA), $[(CH_3)_2CH]_2NLi$, 1.0 M in tetrahydrofuran (THF)/hexanes; and indium chloride ($InCl_3$, 98%) were purchased from Sigma-Aldrich. $InCl_3$ was stored in a glove box purged with N_2 gas. Oleylamine (approximate C18-content 80–90%) was purchased from Acros Organics and purified by vacuum distillation. Oleic acid (>85%) was purchased from TCI. Hydrogen tetrachloroaurate(III) tetrahydrate ($HAuCl_4 \cdot 4H_2O$) was purchased from Kojima Chemicals. Chloroform (99.0%), ethanol (99%), methanol (99.5%) and toluene (99.0%) were purchased from Fujifilm Wako Chemicals.

The size and morphology of each NP were characterized by TEM (HITACHI HT7820) operating at 200 kV. NPs were purified and a drop of solution was drop-casted on carbon-coated Cu grids for TEM. The atomic resolution analytical TEM (JEOL JEM-ARM200CF) observation was operated at an acceleration voltage of 120 kV. A Wiener filter was applied to original TEM-EDX images for noise reduction[41] using DigitalMicrograph Software (Gatan). The crystal structure was confirmed at multiple locations with and without the filter.

HAADF images and LSPR properties of single NP were obtained by EELS combined with monochromated STEM (JEOL JEM-ARM200F). STEM-EELS was conducted at an acceleration voltage of 200 kV. We heated the SiN membrane at 400°C in the STEM experiments to prevent contamination from organic molecules. The crystal structure of $PtIn_2$ at 400°C was confirmed by high-temperature XRD (Figure S10).

The crystalline structure of the NPs at room temperature was characterized by XRD (PANalytical AERIS). More-detailed XRD patterns were obtained by synchrotron X-ray

radiation experiments performed at the SPring-8 BL02B2 (0.5272 Å). *In situ* high-temperature XRD was measured with a furnace (PANalytical X'Pert PRO MPD).

The elemental ratios of the synthesized NPs were determined by EDX (AMETEK Apollo XF) combined with SEM (HITACHI S-4800).

The optical response of LSPR was determined by UV–vis spectroscopy (HITACHI UH5700) with quartz cuvettes.

SERS spectra were obtained with a Laser Raman Microspectroscopy System Nanofinder 30 (Tokyo Instruments Inc.) equipped with a 532-nm laser diode-pumped solid-state laser (SOC J050GS-1H) at an output power of 5.132 mW through a 50× microscope objective (Olympus MPlanFLN) over the range of 590–1819 cm^{-1}. The exposure time was 1 s and not accumulated (only 1×) to avoid heavy damage to rhodamine 6G (R6G). A charge-coupled device camera (1024 px × 255 px) operating at −70°C was used as the detector. To verify that the spectra were reproducible, more than 10 different areas were measured for each sample.

Synthesis of Pt seed NPs

To obtain $PtIn_2$ NPs, Pt seed NPs were synthesized first. Oleic acid (31.5 mmol, 10 mL) and oleylamine (97.2 mmol, 32 mL) were mixed in a 100-mL, three-necked flask; followed by stirring at 220°C in an N_2 atmosphere. $Pt(acac)_2$ (0.5 mmol, 196.68 mg) was added to oleylamine (24.3 mmol, 8 mL) in a 50-mL, three-necked flask; followed by stirring and heating in an N_2 atmosphere. After the $Pt(acac)_2$ was dissolved in the oleylamine, the solution turned bright yellow at ca. 120°C. The mixture was quickly injected into the preheated oleic acid/oleylamine solution. Immediately after injection, the color of the solution changed to blackish brown after 1 min and black after 5 min. The solution was maintained at 220°C for 30 min and cooled at room temperature. Four-fold scale-up was carried out through this method.

Pt seed NPs were well dispersed in chloroform. To remove excess oleic acid and oleylamine, 15 mL of ethanol was added as a poor solvent to 25 mL of crude solution collected with chloroform, followed by centrifugation for 10 min at 9390×*g*, and then stored in chloroform.

Synthesis of $PtIn_2$ NPs

*C*1-$PtIn_2$ NPs were synthesized by reacting Pt seed NPs with In amide complexes in oleylamine under strong basic conditions.[26] The expected reaction is as follows: a strong Brønsted base deprotonates a long-chain primary amine, which reacts with $InCl_3$ to form an In oleylamide.[26]

The *in-situ* formed amide is then reduced or thermally decomposed to react with the Pt seed NPs to form the target product. In this method, the size of the $PtIn_2$ NPs is larger than that of the Pt seed NPs, and the formation of indium oxide during the reaction process is also suppressed because of the absence of oxygen atom in precursors and solvents.

As a strong base, 1.0 M LDA in THF/toluene (3.6 mmol, 3.6 mL) was added to distilled oleylamine (6.1 mmol, 2 mL) in an N_2 gas-filled 20-mL Schlenk tube. THF and toluene were evaporated in advance. With another N_2 gas-filled 20-mL Schlenk tube, distilled oleylamine (9.1 mmol, 3 mL) was injected into $InCl_3$ (0.75 mmol, 165.87 mg) that had been stored in a glove box, followed by sonication at ca. 40°C.

Pt seed NPs were additionally purified for the next step. Pt NPs (0.5 mmol of Pt atoms) were collected in a 100-mL three-necked flask with chloroform; added to distilled oleylamine (30.4 mmol, 10 mL); and sonicated for a few minutes. Then, chloroform was removed by evaporation, additionally dried under vacuum (45 min at 120°C), and heated to 180°C under N_2. LDA–oleylamine solution was injected, after 30 s, followed by injection of $InCl_3$–oleylamine solution. The reaction solution was maintained at 180°C for 2 h and then cooled to room temperature. Although the oleylamine used as a solvent provided the dispersibility of the NPs as a ligand, the dispersibility of the synthesized NPs was improved by injecting 2 mL of oleic acid when the temperature reached 60°C during cooling before opening to the air. Larger-sized $PtIn_2$ NPs were obtained by setting the heating temperature to 240°C to induce the fusion of NPs (Figure 2d).

As is clear from the phase diagram (Figure S3), the region where $PtIn_2$ is stable is narrow. When the reaction conditions differed, Pt_2In_3 and Pt_3In_7 phases were readily evident.

The sizes of the NPs were statistically determined from the TEM images. Size distributions of NPs are shown in Figure S2.

Purification of *C*1-$PtIn_2$ NPs

There were two steps of purification: (1) removal of oleylamine and (2) size separation. In step (1), 20 mL of ethanol was added to 15 mL of a chloroform-diluted sample solution and centrifuged at 9390×*g* for 10 min. In step (2), a small quantity (~5 mL) of ethanol was added to the chloroform dispersion, and the mixture was centrifuged. Centrifugal acceleration was modified depending on the NPs size to be separated. These steps were repeated several times and the target size of the $PtIn_2$ NPs was collected. To prevent a decrease in ligand concentration, oleylamine and oleic acid were added during the purification. $PtIn_2$ NPs were well dispersed in

chloroform and retains absorption at least two years even when stored in dispersed solution after purification.

Synthesis and purification of Au NPs

Oleylamine-capped Au NPs were synthesized as reported.[42] Briefly, oleylamine (8.8 mmol, 2.9 mL) was added to toluene (48 mL) in a 200-mL, three-necked flask; followed by stirring and boiling at ca. 120°C. Then, a solution of $HAuCl_4·4H_2O$ (53.7 mg, 0.13 mmol) dissolved in oleylamine (1.2 mL) and toluene (2.0 mL) were quickly injected. The reaction mixture changed to bright magenta in 5 min and gradually deepened over the course of 25 min. Heating was stopped after 2 h and 17-nm-sized Au NPs were precipitated by adding 100 mL of methanol.

The precipitates were centrifuged at 9390×*g* for 5 min with methanol as a poor solvent. The Au NPs were purified again by redispersion in toluene. Small-sized Au NPs were removed by centrifugation by using only toluene.

***In situ* high-temperature XRD of C-supported $PtIn_2$ NPs**

To prevent interparticle fusion as much as possible, a $PtIn_2$ NPs chloroform dispersion was added into carbon (CABOT Vulcane XC-72) chloroform dispersion and left to stand. After washing with ethanol, the C-supported sample was dried thoroughly and packed in an Al_2O_3 holder. The sample was covered with a readily burnable polymeric film, Prolene Thin-Film (Chemplex), to prevent the sample from blowing off during evacuation.

In high-temperature XRD measurements, a vacuum was first drawn to ca. 7 Pa. Then, the XRD pattern was measured at room temperature, following which the temperature was increased at a constant heating rate of 10°C min^{-1}, and the *in situ* XRD pattern was measured every 100°C from 100°C to 400°C. The measurement was repeated 3 times (ca. 2 h) at 400°C to fully confirm the phase stability at 400°C, the same temperature used in EELS measurements. Finally, XRD measurements were carried out again after the temperature decreased to 40°C. XRD patterns of C-support, Al_2O_3 holder, and prolene are shown by reference measurements under atmospheric conditions at room temperature.

SERS samples prepared by spin-coating

To observe SERS spectra, R6G (mixed with NPs) was spin-coated. Briefly, 0.5×10^{-3} M of R6G (octane/ethanol = 1/1 v/v) was mixed with solutions of $PtIn_2$ NPs or Au NPs to adjust the R6G concentration to 1.25×10^{-4} M. Each colloidal solution was dropped onto cleaned Si wafers

rotating at 2500 rpm for 30 s (no hydrophobic treatment). R6G solution (1.25×10^{-4} M) was spin-coated as a reference.

The quality factors (Q-factors) were evaluated from the measured extinction spectra of the NP dispersion. $Q = \omega / \Gamma$, where ω is the center of the plasmon resonance with a full width at half maximum Γ.

XRD pattern simulation

The XRD pattern of $PtIn_2$ was simulated using CrystalMaker X and CrystalDiffract 6.9 (CrystalMaker Software Ltd.) (Figure S14). The occupancy of Pt at the Pt sites in the $C1$ structure was taken as an independent variable, and the occupancy of In sites was taken as a dependent variable so that the overall molar ratio of Pt : In = 1 : 2 was maintained (Table S1). As shown in Figure S14, the superlattice peaks of $C1$ structure are 111, 200, and 311 from lower angle. For example, the intensity of the 111 peak is smaller for lower Pt occupancy of the Pt sites.

Theory and computational details

The time-dependent Kohn–Sham equation solved in the Scalable Ab-initio Light–Matter simulator for Optics and Nanoscience (SALMON) is as follows (in atomic units):

$$i\frac{\partial}{\partial t}\psi_j(\mathbf{r},t) = \left[-\frac{1}{2}\nabla^2 + v_{\mathrm{nuc}}(\mathbf{r},t) + v_{\mathrm{h}}(\mathbf{r},t) + v_{\mathrm{XC}}(\mathbf{r},t) + v_{\mathrm{ext}}(\mathbf{r},t)\right]\psi_j(\mathbf{r},t), \tag{1}$$

Where $v_{\mathrm{nuc}}(\mathbf{r},t)$, $v_{\mathrm{h}}(\mathbf{r},t)$, $v_{\mathrm{XC}}(\mathbf{r},t)$, and $v_{\mathrm{ext}}(\mathbf{r},t)$ are the nuclear attraction potential, Hartree potential, exchange-correlation potential, and external potential of an applied laser field, respectively. In this study, we adopted the laser pulse, the functional form of which is

$$v_{\mathrm{ext}}(\mathbf{r},t) = -r_v F_t(t), \tag{2}$$

where $F_t(t)$ is the laser field given by

$$F_t(t) = F\sin(\omega t)\sin^2(\pi t/\tau) \qquad (0 < t < \tau), \tag{3}$$

where ω is the laser frequency, and τ determines the laser pulse duration. The electron dynamics under the laser pulse were analyzed by using the Fourier-transformed formula

$$\Delta\rho(\mathbf{r},E) = \frac{1}{F_{\mathrm{re}}}\int\int_0^T [\rho(\mathbf{r},t) - (\mathbf{r},0)]\cdot\left\{1 - 3\left(\frac{t}{T}\right)^2 + 2\left(\frac{t}{T}\right)^3\right\}e^{iEt}dt, \tag{4}$$

where F_{re} is the normalized factor given by

$$F_{\mathrm{re}} = \mathrm{Im}\left[\int_0^T F_t(t)\cdot\left\{1 - 3\left(\frac{t}{T}\right)^2 + 2\left(\frac{t}{T}\right)^3\right\}e^{iEt}dt\right]. \tag{5}$$

We used only the imaginary part because the sin-type wave was used [Eq. (3)]. Then, the imaginary part of $\Delta\rho$ exhibits the spatial configuration of the resonance response of the photoexcited electrons.[4] The Fourier-transformed electric field

$$\Delta\mathcal{E}(\mathbf{r},E) = \frac{1}{F_{\mathrm{re}}}\int \Delta\rho\left(\mathbf{r}',E\right)\frac{\mathbf{r}-\mathbf{r}'}{\left|\mathbf{r}-\mathbf{r}'\right|^3}d\mathbf{r}' , \tag{6}$$

was also analyzed to evaluate the photogenerated electric field around the NPs.

We investigated the photoexcited electron dynamics of large clusters of ca. 600 atoms, Au_{561} and $Pt_{249}In_{432}$, with SALMON.[43] The geometrical structures were determined from bulk crystal data. All the electrons other than the Au $5d^{10}6s^1$ electrons were replaced with effective core potentials obtained by the Troullier–Martins scheme implemented in the fhi98PP program.[44,45] For the exchange–correlation potential, the local density approximation functional was used. The computation box and grid spacing were set to 46 Å × 46 Å × 46 Å and Δx = Δy = Δz = 0.25 Å, respectively. The propagation time was 12.8 fs for the oscillator strength and 20.0 fs for the laser-induced electron dynamics. The applied laser pulse intensity was set to 10^9 W cm^{-2}.

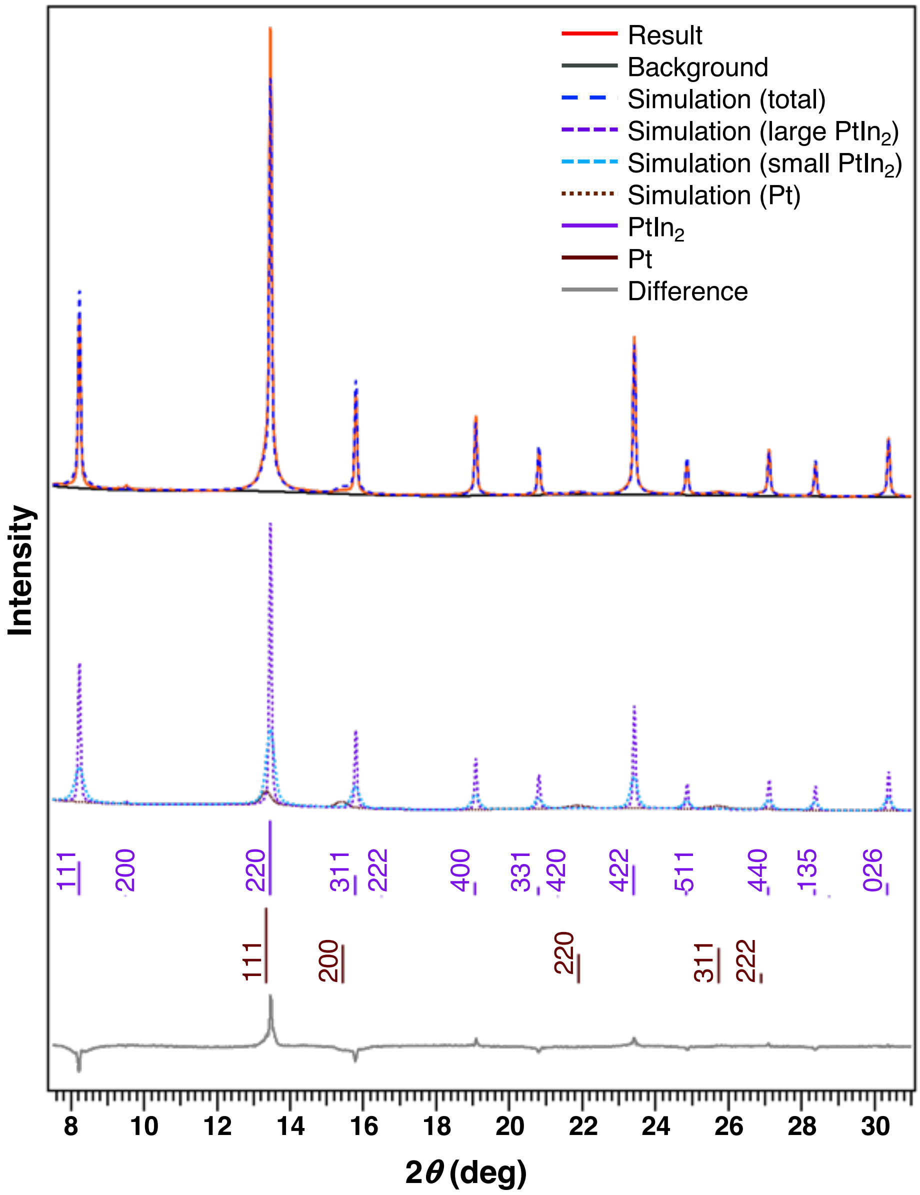

Figure S1. Rietveld refinement of 44-nm $PtIn_2$ NPs. We measured the synchrotron XRD pattern of 44-nm $PtIn_2$ NPs (red, solid line) with $\lambda = 0.527$ Å. We conducted Rietveld analysis by using the standard pattern of Pt and two different patterns of $PtIn_2$ with different sizes obtained from Ref. [32]. Total simulated result (blue dotted line) is the sum of the simulation results for two different sized *C*1-$PtIn_2$ (violet and light blue dotted lines) and Pt (brown dotted line). The reference patterns for $PtIn_2$ (violet solid line, PDF #01-071-5016) and Pt (brown solid line, PDF #00-004-0802) calculated at $\lambda = 0.527$ Å and the difference between the experimentally observed and simulated patterns (gray solid line) are shown. R_{wp} was 6.87% and the goodness-of-fit (GOF) was 3.45. The NPs comprised *C*1-$PtIn_2$ phase (92.6 wt%) with smaller *fcc*-Pt-based crystallites (7.4 wt%).

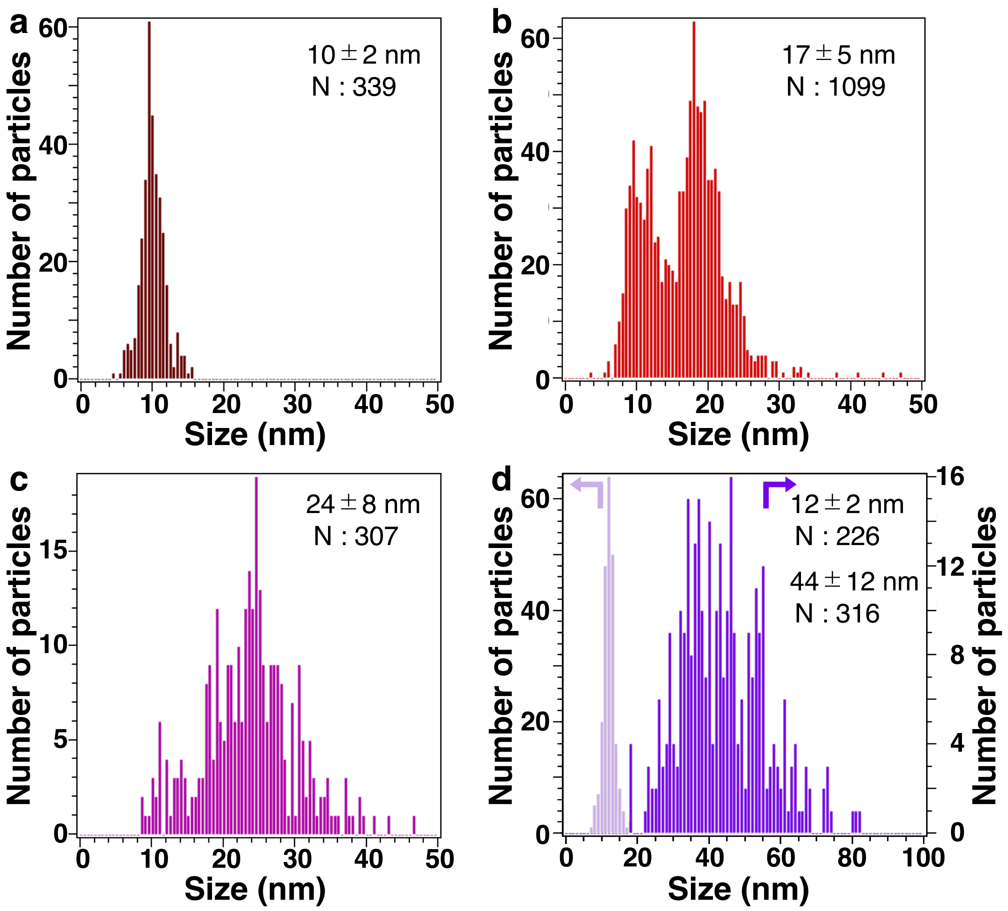

Figure S2. Size distribution histograms of synthesized NPs. Size distribution histograms of a) 10 ± 1 nm Pt seed NPs, b) 17 ± 5, c) 24 ± 8, and d) 44 ± 12 nm $PtIn_2$ NPs obtained from TEM images, corresponding to Figure 2. The smaller NPs in (d), shown in light purple, were 12 ± 2 nm in diameter, slightly larger than the Pt seed NPs. The numbers of particles measured are (a) N = 339, (b) N = 1099, (c) N = 307, and (d) N = 226 for smaller NPs and N = 316 for larger NPs.

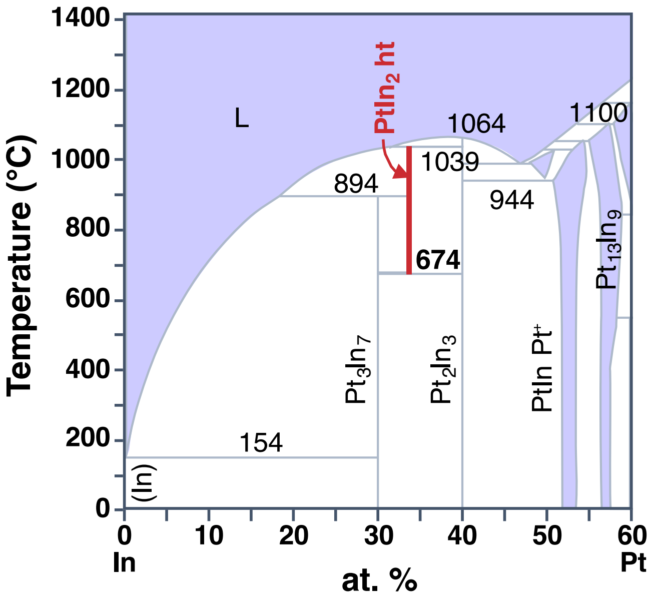

Figure S3. Pt–In phase diagram. We adapted this phase diagram from Ref. [27]. The $PtIn_2$ phase was evident at temperatures greater than 674°C. The $PtIn_2$ phase is over an especially narrow composition range, and readily coexists with the Pt_2In_3 and Pt_3In_7 phases.

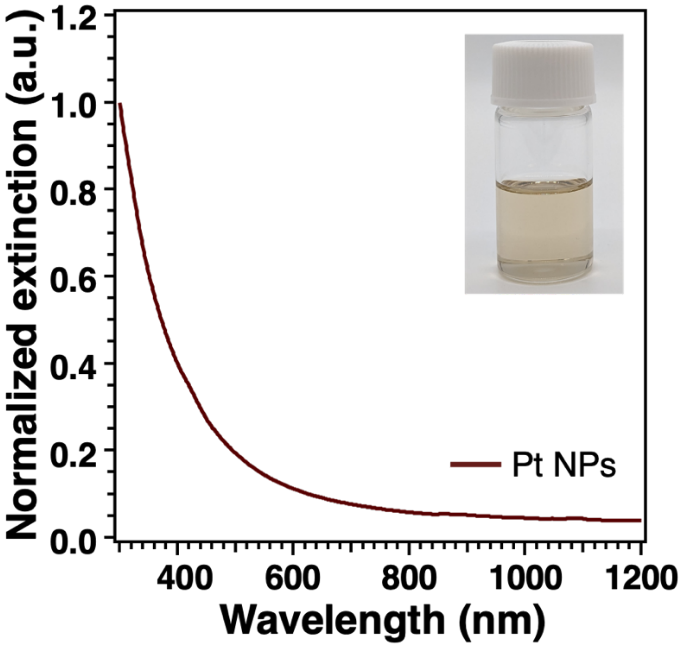

Figure S4. UV–vis–NIR spectrum and photo (inset) of Pt seed NPs.

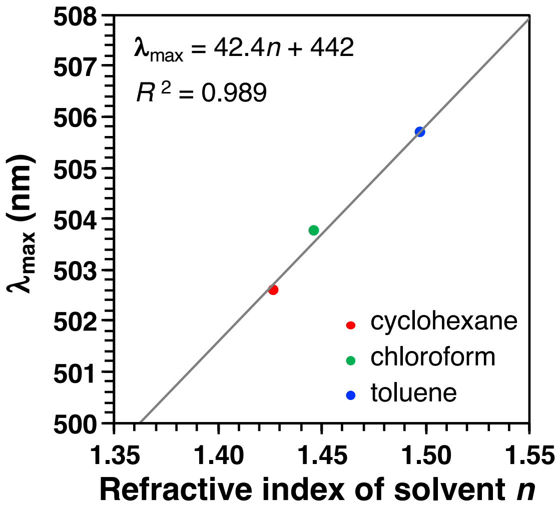

Figure S5. Dependence of λ_{max} of $PtIn_2$ NPs on the surrounding refractive index. We plotted the λ_{max} values of C1-$PtIn_2$ NPs dissolved in cyclohexane (n = 1.4268, red), chloroform (n = 1.4467, green), and toluene (n = 1.4978, blue) against the refractive indices of the solvents. $PtIn_2$ NPs (17 nm) were used because of their high solubility in these solvents. The λ_{max} is proportional to the refractive index of the solvent, which also supports the LSPR feature of C1-$PtIn_2$ NPs. The sensitivity of LSPR λ_{max} to the changes in the surrounding RIU is defined as λ_{max}/RIU = 42.4.

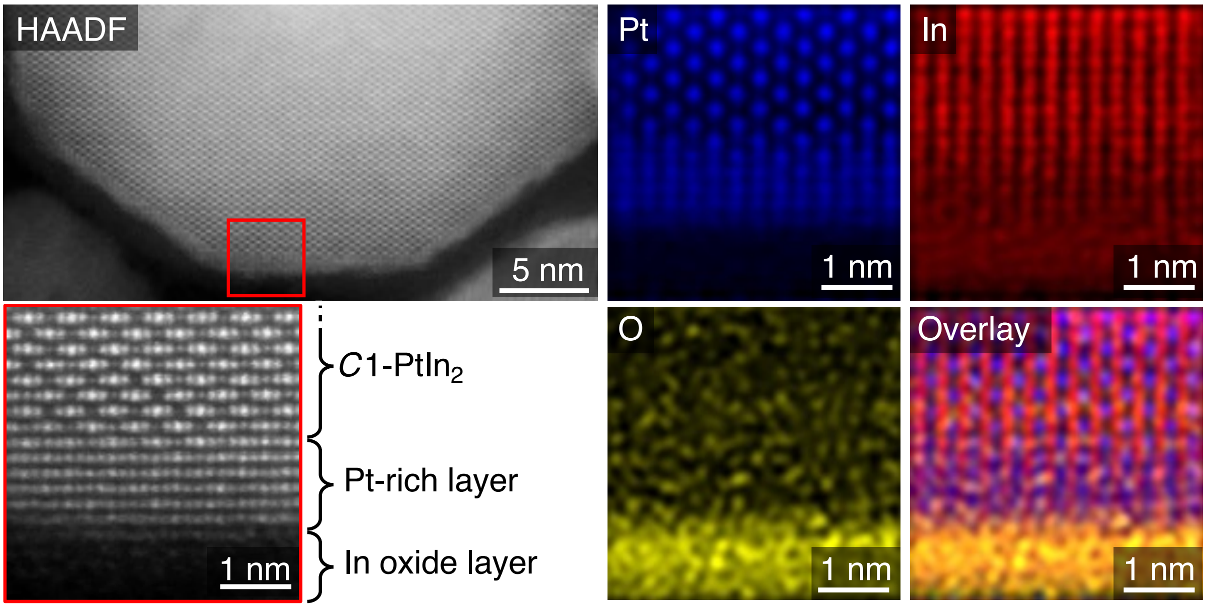

Figure S6. HAADF-STEM and TEM-EDX imaging of $PtIn_2$ NP surface. *C*1-$PtIn_2$ NP was observed from <110> by the HAADF-STEM. The area in red square shows the double-shell structure. TEM-EDX mappings show the distributions of Pt element (blue), In element (red), and O element. Element mapping overlay for Pt, In, and O clearly shows the different compositions of each shell. During the purification and storage, In on the surface of $PtIn_2$ NPs might be gradually oxidized to form indium oxide layers as outer shell and Pt-rich layers as inner shell.

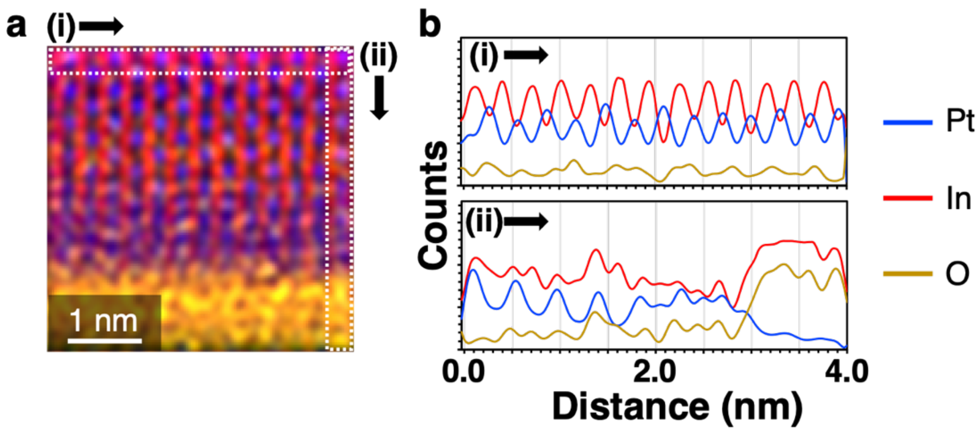

Figure S7. The line-scan EDS curves for Pt (blue), In (red), and O (yellow). a) Two line-scan positions (i, ii) and b) the line-scan curves. As seen in line-scan curves in (i), Pt and In exist alternately. On the other hand, the longitudinal line-scan curves in (ii) clearly show the formation of a double-shell structure at the surface.

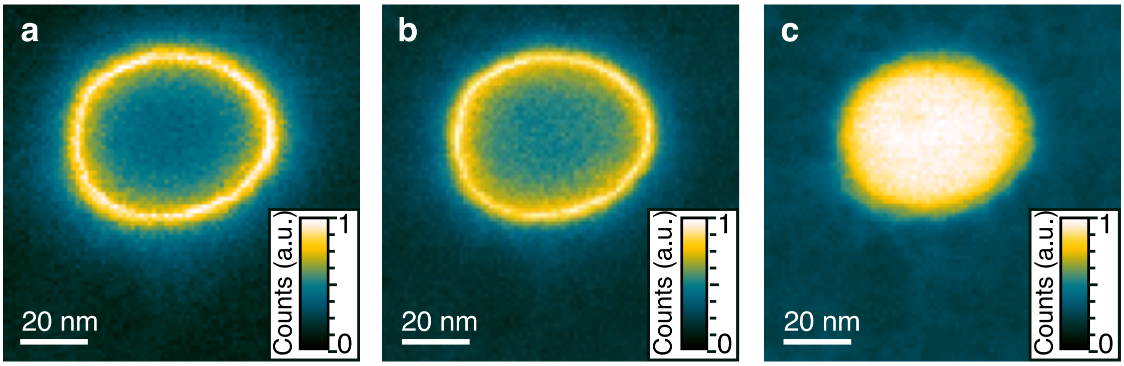

Figure S8. STEM-EELS maps of a single $PtIn_2$ NP. EEL spectra of 44-nm $PtIn_2$ NP exhibit higher-energy losses at a) 4.7–5.1 eV, b) 8.2–8.6 eV, and c) 14.0–14.4 eV. (a) and (b) show the large intensity at the surface of the NP. This corresponds to LSPR at the high energy reported here (i.e., higher-order LSPR), which we consider to be dipole and quadrupole modes. In contrast, interband transitions and/or bulk plasmons (c) corresponded to a large intensity at the center.

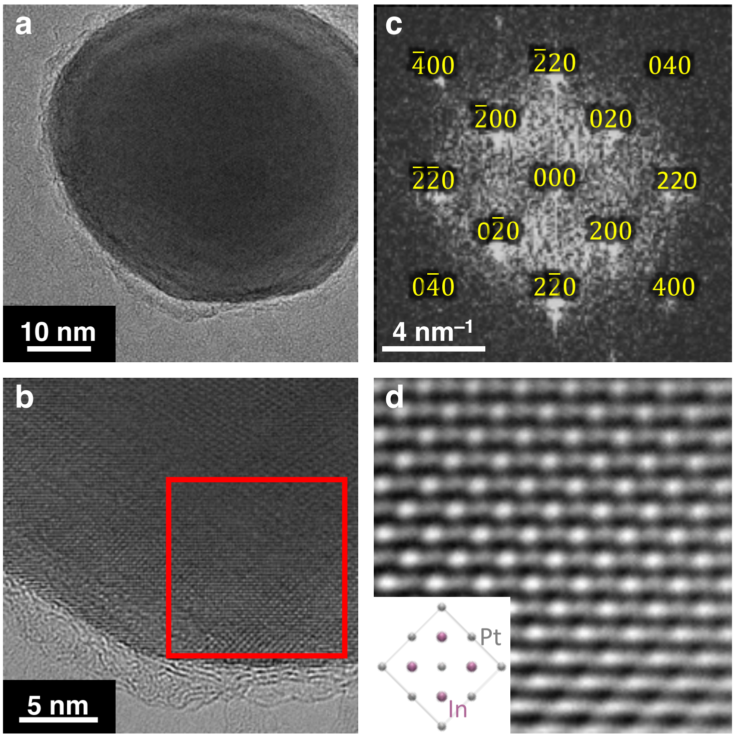

Figure S9. Crystal structure analysis of a single $PtIn_2$ NP by high-resolution TEM. a, b) High-resolution TEM images of a $PtIn_2$ NP measured at 400°C after EELS measurements. c) FFT image of the red square region of (b). The spots corresponding to the 200, 220, and 400 reflections of the *C*1 structure are indicated in yellow. d) Noise-filtered inverse FFT result of (c). The inset in (d) is the crystal lattice of *C*1-$PtIn_2$, where lighter and darker dots correspond to Pt and In atoms, respectively. The atomic arrangement in (d) is similar to that in the inset.

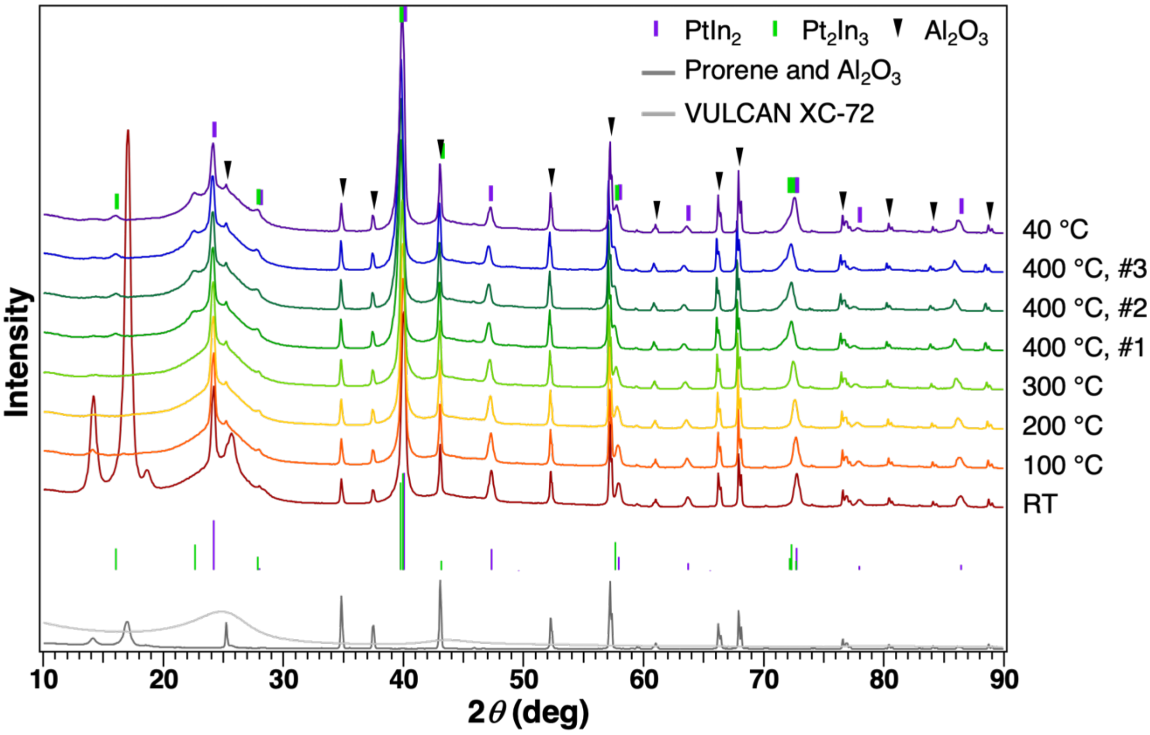

Figure S10. High-temperature XRD measurements of *C*1-$PtIn_2$ NPs supported on carbon. The stability of $PtIn_2$ at high temperatures was investigated every 100°C from room temperature. At room temperature, the peak of the Prolene film used for the cover appeared strongly, but this film burned out at 100°C. We observed the peak that corresponds to $PtIn_2$ at high intensity even at 400°C, although it was slightly shifted towards lower angles due to the thermal expansion of the lattice. At 400°C, we identified a small quantity of Pt_2In_3 in the diffraction pattern as the binary phase with a broader FWHM than that of $PtIn_2$ and did not increase over time. Thus, we propose that nearly unreacted Pt seed NPs contained as minor components reacted with the $PtIn_2$ phase to give the Pt_2In_3 phase. The aforementioned examination strongly supports our proposal that an isolated $PtIn_2$ NP can retain its *C*1 structure even during EELS measurements at 400°C.

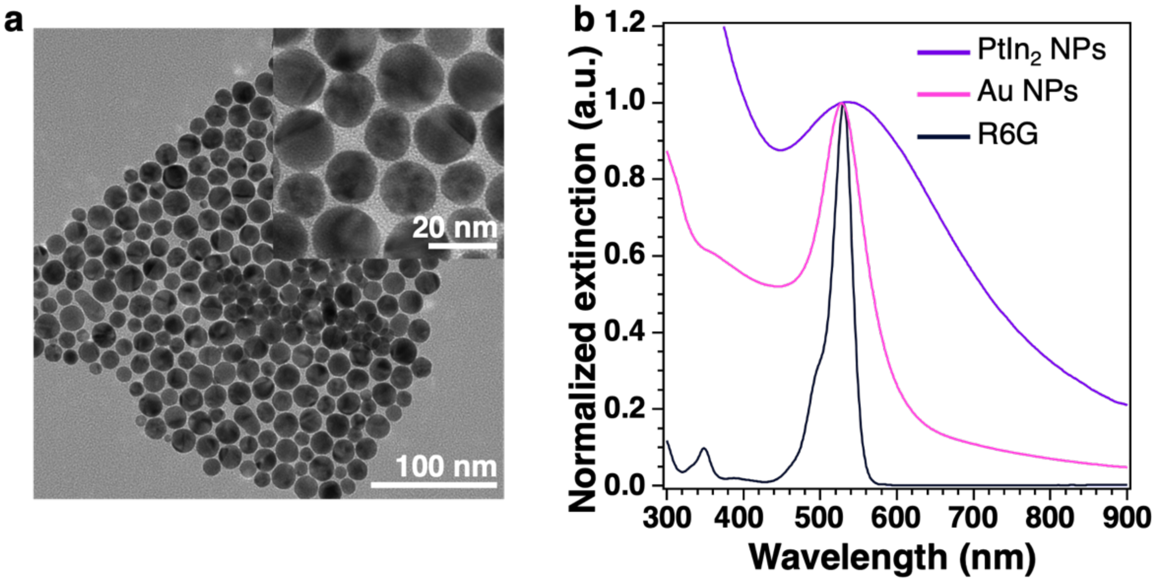

Figure S11. TEM images of Au NPs and optical properties of SERS samples. a) TEM images of Au NPs used for SERS. Nearly spherical NPs are 17 ± 4 nm in diameter. b) Normalised extinction spectra of 24-nm *C*1-$PtIn_2$ NP dispersion in chloroform (violet), Au NP dispersion (pink), and R6G solution (black); with λ_{max} at 534, 527, and 530 nm, respectively. The quality factor of this resonance can be described by $Q = \omega / \Gamma$ where ω is the centre of the plasmon resonance with a FWHM of Γ. Q_{PtIn2} = 2.80 was smaller than Q_{Au} = 7.98.

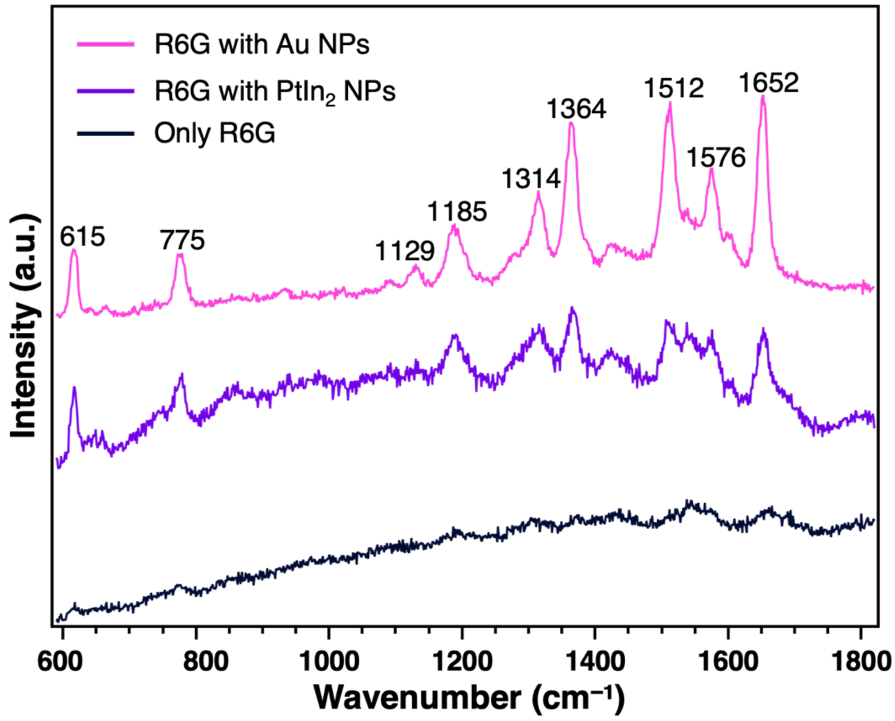

Figure S12. SERS measurements. SERS spectra of R6G (black), R6G with 24-nm $PtIn_2$ NPs (violet), and R6G with 17-nm Au NPs (pink) on a Si wafer with a 532-nm laser as the excitation light source. Although the R6G sample exhibited almost no clear peaks, both NPs drastically enhanced the SERS signals caused by the electric field enhancement effect of LSPR. As expected from the Q values, the SERS enhancement by the Au NPs was stronger than that by the $PtIn_2$ NPs.

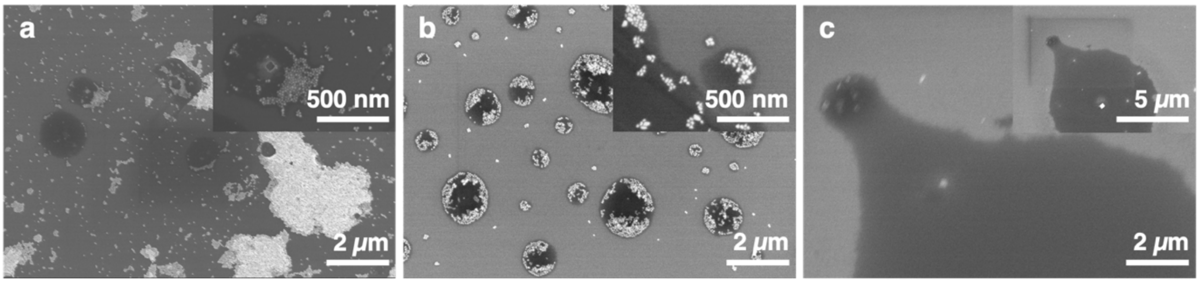

Figure S13. SEM image of the substrate used for surface-enhanced Raman measurements: a) Rhodamine 6G (R6G) with Au NPs, b) with $C1$-$PtIn_2$ NPs and c) only R6G on Si wafer.

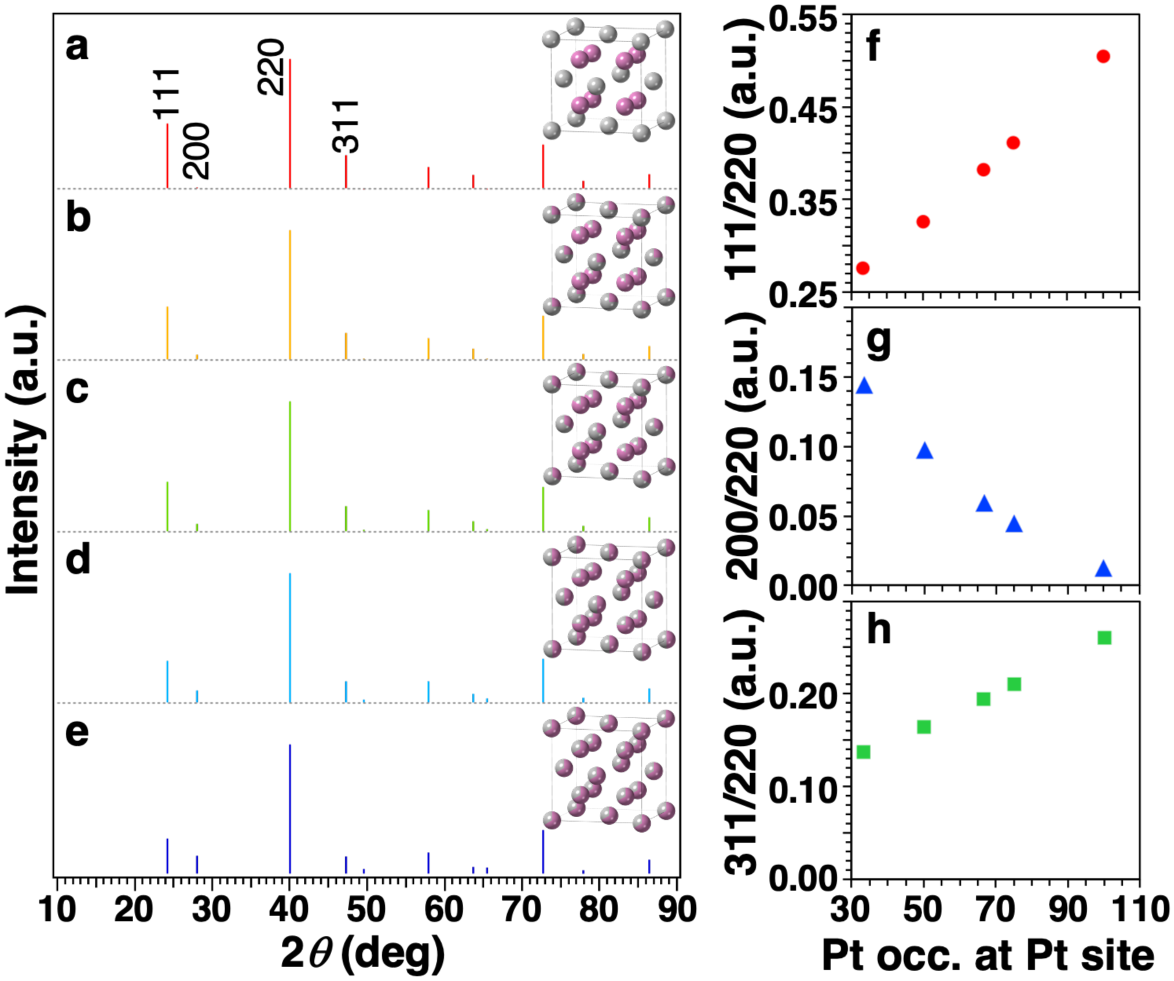

Figure S14. Simulated XRD patterns. XRD patterns were simulated for Pt site occupancies of a) 100 %, b) 75.0 %, c) 66.6%, d) 50.0%, and e) 33.3% while keeping the overall molar ratio Pt : In = 1 : 2 (see Table S1). 220 peak intensity was taken as 100%. 220 peak intensity was set as 100%. Pt occupancy at Pt sites vs. f) 111/220 ratio, g) 200/220, and h) 311/220 of XRD simulation results.

Table S1. Occupancies for XRD simulation.

		Pt occ. at Pt site	In occ. at Pt site	Pt occ. at In site	In occ. at In site
**(a)**	100.0%	1.0000	0.0000	0.0000	1.0000
**(b)**	75.0%	0.7500	0.2500	0.1250	0.8750
**(c)**	66.6%	0.6666	0.3334	0.1666	0.8334
**(d)**	50.0%	0.5000	0.5000	0.2500	0.7500
**(e)**	33.3%	0.3333	0.6667	0.3333	0.6667

References

[41] L. D. Marks, *Ultramicroscopy* **1996**, *62,* 43–52.

[42] H. Hiramatsu, F. E. Osterloh, *Chem. Mater.* **2004**, *16*, 2509–2511.

[43] M. Noda, S. A. Sato, Y. Hirokawa, M. Uemoto, T. Takeuchi, S. Yamada, A. Yamada, Y. Shinohara, M. Yamaguchi, K. Iida, I. Floss, T. Otobe, K.-M. Lee, K. Ishimura, T. Boku, G. F. Bertsch, K. Nobusada, K. Yabana, *Comput. Phys. Commun.* **2019**, *235*, 356–365.

[44] N. Troullier, J. L. Martins, *Phys. Rev. B* **1991**, *43*, 1993–2006.

[45] M. Fuchs, M. Scheffler, *Comput. Phys. Commun.* **1999**, *119*, 67–98.
